# Clinical Characteristics of Gait Disturbance in Charcot-Marie-Tooth Disease and Future Directions in Physical Therapy

**DOI:** 10.7759/cureus.85581

**Published:** 2025-06-08

**Authors:** Kazuto Kikuchi

**Affiliations:** 1 Physical Therapy, Akita Rehabilitation College, Akita, JPN

**Keywords:** charcot-marie-tooth disease, gait characteristics, gait disturbance, physical therapy, rehabilitation，

## Abstract

Charcot-Marie-Tooth disease (CMT) is a hereditary and progressive peripheral neuropathy affecting both motor and sensory nerves. It is among the most common inherited neuropathies and is primarily classified into demyelinating (type 1) and axonal (type 2) forms based on motor nerve conduction velocity, with over 100 genetic subtypes identified. Due to this genetic and clinical heterogeneity, the onset, severity, and degree of motor and sensory impairments vary widely among individuals.

One of the hallmark manifestations of CMT is gait disturbance. As the disease progresses, individuals often develop foot drop and foot deformities such as pes cavus and equinus, leading to a significant decline in gait function. This results in limitations in activities of daily living, increased risk of falls, reduced social participation, and decreased quality of life. Currently, no curative treatment exists for CMT. Management focuses on symptomatic interventions, including orthotic support, surgical procedures, and physical therapy. While physical therapy may improve muscle strength and physical function, the quality of evidence remains moderate, and no standardized rehabilitation protocols have been firmly established. Tailored physical therapy programs are considered essential for effective intervention.

Given these challenges, the need for quantitative and objective assessment of gait disturbances in CMT has become increasingly important. Conventional clinical scales, such as the CMT Neuropathy Score, rely heavily on subjective grading and offer limited value in detailed gait analysis. In contrast, recent advances in motion analysis - such as three-dimensional gait analysis, ground reaction force measurement, and wearable sensors - have provided more precise assessments. However, issues related to standardization and clinical applicability remain unresolved. This review aims to summarize the neuropathology and clinical characteristics of gait disturbances in CMT, discuss current gait assessment methodologies, explore physical therapy strategies, and highlight the need for quantitative evaluation and future research directions in rehabilitation for this patient population.

## Introduction and background

Charcot-Marie-Tooth disease (CMT) is a hereditary and progressive peripheral neuropathy that affects both motor and sensory nerves. It is one of the most common inherited neuropathies. Based on motor nerve conduction velocity, CMT is primarily classified into demyelinating (type 1) and axonal (type 2) forms, with over 100 genetic subtypes identified to date [1-5]. Due to its genetic and clinical heterogeneity, there is substantial variability among individuals in terms of age of onset, disease severity, and the extent of motor and sensory impairment.

The prevalence of CMT is estimated at approximately 20 per 100,000 population, making it the most common disorder among 17 evaluated neuropathies. A pooled analysis of multiple studies estimated the prevalence at 17.69 per 100,000 (95% CI: 12.32-24.33), highlighting its relatively high frequency compared to other neurological disorders [6].

Gait disturbance is one of the hallmark features of CMT. As the disease progresses, patients frequently develop dorsiflexion weakness (i.e., foot drop) and foot deformities such as pes cavus and equinus, which significantly impair gait function. These motor deficits result in reduced ability to perform activities of daily living (ADL), an increased risk of falls, restricted social participation, and ultimately, diminished quality of life (QOL) [7].

Currently, no curative treatments exist for CMT. Management is largely supportive and includes orthotic devices, surgical interventions, and physical therapy. Systematic reviews have indicated that physical therapy may improve muscle strength and physical function; however, the quality of evidence remains moderate, and a standardized rehabilitation protocol grounded in high-level scientific evidence has yet to be established [8]. Moreover, the need for individualized physical therapy programs tailored to each patient's functional status has been increasingly emphasized.

A systematic review by Sman et al. analyzed 13 studies and reported that physical interventions, including resistance training and aerobic exercise, may enhance muscle strength, physical function, and ADL. Notably, lower limb resistance training over 12 weeks led to improvements in ankle plantarflexor strength in one study, while aerobic exercise using a cycle ergometer increased peak oxygen uptake in another. Despite these promising findings, many studies were limited by small sample sizes, lacked statistical power, and exhibited methodological heterogeneity and inconsistency in outcome measures [8].

Similarly, Corrado et al. reviewed 10 studies on conservative rehabilitative approaches, including exercise therapy, physical therapy, and orthotic use, and found that improvements in muscle strength and endurance may lead to enhanced ADL performance. Among these, progressive resistance training was reported to slow muscle atrophy and support the maintenance of walking ability and functional independence. However, evidence regarding the efficacy of orthoses remains inconclusive, and scientific support for their use is still limited [9].

A separate systematic review and meta-analysis focusing on the effectiveness of ankle-foot orthoses (AFOs) included 15 studies and found that AFOs may influence spatiotemporal, kinematic, and kinetic gait parameters, such as walking speed, stride length, and joint motion at the ankle, knee, and hip, as well as ankle joint moments. Nevertheless, among the eight studies eligible for meta-analysis, no statistically significant improvements were observed. Moreover, data on postural stability and balance were insufficient for quantitative analysis. The small sample sizes, variation in AFO design, and diversity in assessment methodologies were cited as key limitations affecting the consistency of findings [10].

These findings support the potential benefits of physical rehabilitation for individuals with CMT but also highlight the moderate quality of evidence and the absence of standardized intervention protocols. Future research must focus on identifying optimal exercise modalities, including type, intensity, frequency, and duration, as well as assessing their long-term effects. Given the rarity of CMT, larger and more methodologically rigorous clinical trials are essential to clarify the effectiveness of interventions such as AFOs. Personalized rehabilitation strategies remain crucial, with the ultimate goal of developing evidence-based therapeutic approaches that support functional preservation and improve quality of life in patients with CMT.

In this context, the quantitative and objective assessment of gait disturbances in individuals with CMT is gaining importance. Traditional clinical tools, such as the Charcot-Marie-Tooth Neuropathy Score, rely on ordinal scales (0-4) and involve subjective judgments, which limit their utility in detailed gait analysis. In recent years, advanced technologies, including three-dimensional motion analysis, ground reaction force measurement, and wearable sensors, have been adopted to enable more precise gait assessments. However, challenges related to standardization and clinical integration of these techniques remain.

This review aims to provide a comprehensive overview of the neuropathology, clinical features, gait assessment methods, and rehabilitation strategies for gait disturbances in CMT. Additionally, we highlight emerging techniques for quantitative gait analysis and propose future directions in this field.

## Review

Methods

Study Design: Literature Review

Study Eligibility Criteria: The review included studies based on defined eligibility and exclusion criteria. All primary research articles, regardless of study design, were considered, including systematic reviews, meta-analyses, randomized controlled trials (RCTs), quasi-experimental studies, and case studies. Studies were included if they investigated physical therapy or rehabilitation interventions in the context of CMT. Reports published in languages other than English and conference abstracts were excluded. Only articles retrieved from the PubMed database were included in this review. Studies were grouped according to intervention types and outcome domains to facilitate the synthesis and interpretation of results.

Search Strategy: A systematic literature search was conducted using the PubMed database between late April and early May 2025. The aim was to comprehensively identify studies addressing physical therapy and rehabilitation interventions, as well as the characteristics of gait disturbances in individuals with CMT. Search terms included both MeSH (Medical Subject Headings) and free-text keywords such as: "Charcot-Marie-Tooth Disease," "Hereditary Motor and Sensory Neuropathy," "Rehabilitation," "Physical Therapy," "Orthotic Devices," "Gait," "Walking Analysis," "Exercise Therapy," "Orthotic Therapy," "Surgery," "Robotics," and "Virtual Reality". Eligible articles were limited to original research papers, systematic reviews, meta-analyses, randomized controlled trials, quasi-experimental studies, and case reports written in English. Non-English literature and conference proceedings were excluded. Search queries were adapted for the PubMed database to ensure optimal retrieval and reproducibility. Detailed search strings are provided in Appendix 1.

Neuropathological features of CMT

A thorough understanding of the neuropathological features of CMT is essential for accurate diagnosis and the development of effective therapeutic strategies. Histopathological examination, in particular, plays a vital role in identifying disease subtypes and guiding appropriate treatment approaches. Furthermore, the integration of genetic diagnostics with pathological findings may improve prognostic predictions and facilitate the development of targeted therapies.

CMT is a hereditary peripheral neuropathy characterized by abnormalities in either the myelin sheath or the axon itself, resulting in impaired nerve conduction velocity.

Myelin Abnormalities

CMT type 1 is caused by genetic mutations that disrupt the formation and maintenance of the myelin sheath [11]. These defects lead to abnormal thickening of the myelin surrounding peripheral nerve fibers. Characteristic pathological features include “onion bulb” formations - concentric layers of redundant myelin - and abnormal Schwann cell proliferation [2]. These structural abnormalities significantly reduce nerve conduction velocity and result in both motor and sensory dysfunction.

Axonal Degeneration

CMT type 2 is primarily associated with axonal pathology. Unlike type 1, there is no evidence of myelin sheath abnormalities. Instead, degeneration and shrinkage of the axons themselves are the primary pathological findings [3]. Histopathological examination typically reveals a reduction in axonal density and various structural abnormalities in the axonal components [4]. These changes impair peripheral nerve function, leading to progressive muscle weakness and sensory deficits.

Shared Pathological Features

Despite the differences between demyelinating and axonal forms, a common feature across CMT subtypes is the degeneration of both myelin and axonal structures within peripheral nerves [5]. These degenerative changes contribute to decreased nerve conduction velocity and result in functional impairments affecting both motor and sensory systems.

Clinical manifestations

The age of symptom onset in CMT varies widely among patients, with a mean age of 25.1 ± 19.9 years (range: 0-77 years; median: 20 years). A substantial proportion of individuals report initial symptoms during childhood, particularly before the age of 15. The most commonly reported early symptoms include gait disturbances, foot deformities, and sensory abnormalities such as pain and numbness. Pain is most frequently localized to the ankle, plantar surface, lower back, and knee, in that order. Numbness typically affects the fingertips and toes. In some cases, foot drop may precede awareness of other symptoms [6].

Muscle Weakness and Atrophy

Muscle weakness and atrophy typically appear in the distal limbs during the early stages of the disease. In the lower extremities, the tibialis anterior and gastrocnemius muscles are frequently involved, resulting in impaired dorsiflexion and plantarflexion of the ankle and contributing to gait disturbances [12]. In the upper extremities, the thenar and intrinsic hand muscles are commonly affected, leading to reduced grip strength and manual dexterity [13].

Sensory Disturbances

Patients exhibit impairments in both superficial and deep sensation, particularly in distal regions. Notably, a significant reduction in tactile sensitivity on the plantar surface compromises proprioception and the ability to perceive ground contact during ambulation [12]. Similarly, tactile and thermal sensations in the palms and fingers are often diminished, leading to difficulty with fine object discrimination [13]. Impaired joint position sense in the knees and ankles further contributes to abnormal limb positioning during gait [14]. Reduced nerve conduction velocity exacerbates sensory deficits and is a key electrophysiological marker of peripheral neuropathy in CMT.

Muscle Cramps

Painful muscle cramps are commonly reported in the lower limbs, especially in the gastrocnemius and tibialis anterior muscles, as well as in the thenar and intrinsic hand muscles. These cramps can occur abruptly, last from several seconds to minutes, and significantly impact QOL [15]. They are believed to result from impaired neural input due to peripheral nerve damage, leading to abnormal muscle contractions and disrupted motor control [16].

Reduced Deep Tendon Reflexes

As CMT progresses, deep tendon reflexes, particularly at the knees and ankles, often become diminished or absent [17]. In CMT type 1, demyelination slows nerve conduction, attenuating the patellar and Achilles tendon reflexes. In contrast, CMT type 2 is characterized by axonal degeneration, and reflexes may remain relatively preserved, reflecting the differing underlying pathophysiology [2].

Pathophysiology and mechanisms of gait disturbance

In CMT, peripheral nerve dysfunction disrupts normal nerve conduction, resulting in both motor and sensory deficits. These impairments manifest as muscle weakness, sensory loss, and impaired neuromuscular coordination, all of which significantly compromise gait function.

CMT type 1 is primarily a demyelinating neuropathy, characterized by markedly reduced nerve conduction velocities. In contrast, CMT type 2 involves axonal degeneration, with relatively preserved conduction velocity but more pronounced distal muscle atrophy and weakness.

These pathological processes lead to progressive, symmetrical muscle wasting, beginning in the distal lower limbs and feet. Deep tendon reflexes are often diminished or absent, and sensory disturbances are common. A key feature of gait dysfunction in CMT is foot drop, typically resulting from tibialis anterior weakness, which often leads to a compensatory steppage gait. Muscle wasting is most pronounced in the distal thighs and lower legs, creating the classic “inverted champagne bottle” appearance. Marked atrophy of muscles innervated by the peroneal nerve further contributes to the high-stepping gait pattern.

Weakness of the intrinsic foot muscles can lead to pes planus, while impaired dorsiflexion combined with proprioceptive deficits significantly increases the risk of falls. Additionally, reorganization of motor units and muscle fiber-type transformation diminishes muscular endurance, thereby limiting the ability to sustain ambulation [7].

Disruption in the integration of central and peripheral nervous system signals, along with impairments in feedback control mechanisms, further compromise postural control and adaptive gait responses. Weakness of proximal muscles such as the quadriceps and triceps surae further reduces gait efficiency. Impaired intermuscular coordination often results in awkward, arrhythmic gait patterns [12]. Nerve conduction abnormalities destabilize the temporal structure of the gait cycle, particularly delaying the transition between the stance and swing phases. Due to diminished proprioceptive input, patients increasingly rely on visual cues to maintain balance, frequently adopting a widened base of support. As a result, ambulation becomes particularly challenging in dimly lit environments.

Assessment of gait impairment in CMT

Clinical Manifestations: Gait Disturbances and Their Impact

The clinical presentation and rate of disease progression in CMT vary by genetic subtype, with notable differences in motor function between CMT types 1 and 2. CMT type 1, a demyelinating subtype, typically exhibits a slow and gradual progression, whereas CMT type 2, characterized by axonal degeneration, often presents with more severe functional impairments from an earlier age. Age is a key determinant of disease impact; as patients grow older, declines in muscle strength and balance become more pronounced. The age of onset also influences gait patterns and the rate of functional deterioration. In CMT type 2, triceps surae weakness is especially prominent, leading to significantly diminished ankle push-off force and power generation during the stance phase compared to CMT type 1. This contributes to the early loss of normal gait mechanics, particularly the ankle dorsiflexion moment.

As a result, steppage gait frequently emerges in the early stages of the disease, further reducing gait efficiency and increasing susceptibility to fatigue. These gait disturbances severely compromise ADL and QOL, often resulting in restrictions in outdoor mobility, heightened fear of falling, and psychological consequences such as depression. The CMT Pediatric Scale consistently demonstrates higher scores in patients with CMT type 2 than in those with CMT type 1, reflecting more severe disability [18].

Assessment Methods

A study of 20 CMT patients evaluated the reliability of kinematic and electromyographic data using a multi-task gait protocol. Many gait parameters during natural walking showed high test-retest reliability. In particular, the Toe-Heel Score (THS) demonstrated excellent reliability (Intraclass Correlation Coefficient (ICC) = 0.95) and a low standard error of measurement (SEM = 2.7°), indicating its value as a sensitive marker of distal muscle function. Furthermore, more challenging tasks such as toe- and heel-walking correlated strongly with clinical CMT severity scores, enabling more nuanced assessments aligned with disease progression [19].

These findings underscore the utility of gait analysis in functionally assessing CMT patients and highlight the importance of selecting appropriate tasks and parameters. The 6-Minute Walk Test (6MWT) and the StepWatch® Activity Monitor (SAM) (Modus Health, Edmonds, USA) were evaluated in a cohort of 168 individuals with CMT. The 6MWT showed excellent reliability and strong correlations with standard clinical outcome measures. Meanwhile, the SAM demonstrated robust associations with QOL, supporting its use as a complementary tool in functional assessment. Collectively, these tools have been identified as promising metrics in rehabilitation research [20].　

Three-dimensional gait analysis has revealed characteristic gait alterations across varying levels of disease severity. In patients with moderate impairment, decreased gait speed, reduced cadence, and altered joint kinematics were observed. Notably, knee and ankle joint abnormalities became more pronounced with disease progression. These results suggest that gait impairments in CMT type 1 are not solely attributable to muscle weakness, but are also influenced by compensatory motor strategies and limited joint mobility [21].

A study involving 53 CMT patients demonstrated significant correlations between the Charcot-Marie-Tooth Neuropathy Score (CMTNS) and multiple functional performance tests, including the 6MWT, 10-Meter Walk Test (10MWT), Walk-12 questionnaire, and balance assessments such as the Berg Balance Scale (BBS) and the Short Physical Performance Battery (SPPB). These findings validate the clinical utility of these assessments in evaluating motor function in individuals with CMT type 1 [22].

In a case study of a 55-year-old female with CMT, the effects of customized orthopedic footwear were evaluated using both the GAITRite® system (CIR Systems, Inc., Franklin, USA) and clinical assessments. Following intervention, improvements in walking speed and plantar contact area were objectively documented, alongside complete resolution of pain and fall episodes. These objective findings were consistent with the patient's subjective improvements, suggesting that properly designed orthopedic footwear may effectively enhance gait function in CMT patients [23].

Limitations and Challenges in Gait Analysis

Despite their precision, advanced tools such as 3D motion capture systems present practical limitations in routine clinical settings, including the need for specialized facilities, trained personnel, and complex data processing. Moreover, discrepancies in the parameters measured across different gait assessment tools underscore the necessity for standardization. For long-term monitoring, considerations must extend beyond reliability and validity to include cost-effectiveness and feasibility in real-world settings.

Management of gait impairment in CMT

Gait impairment in CMT is progressive and currently lacks curative treatment, making non-pharmacological management, particularly rehabilitation, critically important. This section discusses conventional rehabilitation strategies, the application of emerging technologies, case and research reports, and future directions.

Rehabilitation Approaches

Gait disturbances in CMT are progressive in nature, and in the absence of curative treatments, physical therapy plays a critical role in maintaining functional capacity and improving QOL. As a foundational intervention, physical therapy aims to slow the progression of muscle weakness and maximize residual functional abilities. Targeted strengthening of ankle dorsiflexors and hip musculature, lower limb stretching, and postural control exercises have all been shown to be effective.

A randomized controlled trial involving 14 patients with CMT demonstrated that a 12-week home-based program combining multisensory balance training and proximal muscle strengthening significantly improved balance and gait function, with moderate improvements in postural stability. The intervention was reported to be both feasible and safe for home implementation [24]. A prospective, multicenter, single-blind randomized trial involving 53 outpatients found that those receiving treadmill training in combination with stretching and proprioceptive stimulation showed greater improvements in gait and balance function than those receiving stretching and proprioceptive training alone. These findings support the safety and efficacy of exercise regimens that incorporate treadmill training [25]. A 12-week aerobic exercise program conducted at local gyms resulted in improved peak VO₂ in both the CMT and control groups, with a moderate effect size observed specifically in the CMT cohort [26].

A systematic review of six RCTs involving 214 participants found moderate-quality evidence that ankle dorsiflexor strengthening may slow the progression of muscle weakness in children with CMT. However, the quality of evidence for other outcomes, such as function, QOL, and endurance, was low to very low. Therefore, current evidence supports exercise therapy primarily for pediatric muscle strength preservation [27]. A pilot study on home-based resistance training in patients with CMT revealed a significant increase in left-sided hip flexor strength, but no improvements in walking speed or endurance. No adverse events were reported, and adherence was high; however, due to the limited functional benefits observed, the protocol was deemed suboptimal. The findings suggest the need for more refined patient stratification in future trials [28].

Following a protocol of treadmill training, stretching, breathing, and proprioceptive exercises (TreSPE), improvements in ankle joint angles and six-minute walk distance were observed; however, these gains diminished to baseline levels within six months, suggesting that rehabilitation may need to be administered biannually for sustained benefit [29]. Overall, exercise programs incorporating treadmill training have been shown to be both effective and safe in improving gait and balance in individuals with CMT. Nonetheless, these effects tend to be short-lived, indicating the need for long-term, individualized interventions and periodic reassessments. While evidence supports the role of exercise therapy in attenuating the progression of ankle muscle weakness in children, data regarding other clinical outcomes remain limited. Thus, continued and tailored physical therapy is recommended.

Orthotic therapy is commonly employed to address ankle instability and foot drop. Ankle-foot orthoses (AFOs), particularly those made of lightweight and flexible materials, enhance comfort and fit during ambulation. In patients with sensory deficits, external stimuli provided by orthoses may contribute to improved balance control. AFOs are routinely prescribed to compensate for both foot drop and ankle instability.

A systematic review reported that AFOs may influence walking speed, ankle torque, energy expenditure, and balance indices. Some studies demonstrated statistically significant improvements in ankle moments, energy efficiency, and balance measures. However, many studies failed to show significant changes in gait or balance parameters, and the overall findings remain inconsistent. Variability in orthosis type, outcome measures, and patient characteristics likely contributed to the heterogeneity of results. Therefore, a clear conclusion regarding the general efficacy of AFOs cannot be drawn. Nevertheless, clinically meaningful improvements in specific gait parameters may be achievable when orthoses are tailored to individual patient needs [10].

One study evaluated the use of custom 3D-printed ankle braces and found significant improvements in single-leg stance balance and foot pain. Participants also reported high levels of comfort and satisfaction. However, some users experienced difficulties with donning and individual fit, indicating the need for further device refinement and larger-scale trials [30].

While AFOs do not facilitate neural recovery, they may contribute to improved gait stability and mobility. However, such evidence is primarily based on case reports [31].

Conversely, negative findings have also been reported. For instance, the use of night splints to stretch plantarflexor muscles failed to yield significant improvements in ankle dorsiflexion range of motion (ROM) or muscle strength. After six weeks of use, changes in ROM were minimal and not statistically significant, and no meaningful changes in strength were observed. Thus, night splinting appears ineffective for enhancing ankle function in this context [32].

In several small-scale studies, no significant effects were observed for interventions such as exercise therapy, creatine supplementation, or orthotic use. At present, there is a lack of sufficiently powered RCTs, underscoring the need for large-scale clinical trials using validated outcome measures [33].

Functional electrical stimulation (FES) has been reported as a promising intervention for correcting foot drop. Timed electrical stimulation applied to the tibialis anterior muscle may enhance gait symmetry and efficiency. This technique targets the underlying neuromuscular dysfunction and offers dynamic assistance during walking.

Application of Emerging Rehabilitation Technologies

Recent advancements in rehabilitation technologies have introduced novel approaches to managing gait impairment in CMT. Robot-assisted gait training (RAGT) enables the repetitive practice of physiologically appropriate gait patterns, aligning with motor learning theories. RAGT has shown promise in improving gait function in individuals with neurological disorders, including CMT. Specifically, end-effector-type robotic devices such as the MorningWalk® (Curexo, Seoul, South Korea) have demonstrated potential benefits in enhancing gait speed and muscle activation patterns by providing body weight support and reducing fall risk. Patients with peripheral neuropathies, including CMT, have successfully completed RAGT programs, with reported improvements in motor function and gait performance [34].

An RCT involving 14 patients with CMT type 1 compared a 12-week balance training intervention group with a control group maintaining usual activity. The intervention included fall prevention education, multisensory balance training, and pelvic muscle strengthening, performed three times per week under physical therapist supervision. The training group exhibited significant improvements in balance and gait function as measured by postural assessment systems. However, improvements in lower limb strength were inconsistent [24].

The integration of virtual reality (VR) technologies has also gained attention as a motivational and repetitive training method for chronic neurological diseases like CMT. VR-based rehabilitation may enhance patients' responsiveness to dynamic environments and potentially reduce fall risk through improved sensorimotor interaction. Wearable devices equipped with accelerometers and inertial sensors now allow for real-time collection and analysis of gait data in daily life. This technology supports objective evaluation of interventions and enables remote monitoring. In patients with CMT type 1, wearable sensors demonstrated strong correlations between gait speed and balance indicators with clinical assessments [35]. Such innovations offer a promising avenue for extending rehabilitation into home-based settings and enhancing personalized therapy approaches.

Evidence-Based Interventions and Future Perspectives

High-level evidence for the management of gait impairment in CMT remains limited. As of May 2025, a review of the evidence regarding interventions for gait and functional impairments in CMT identified six Level I studies, including systematic reviews (SRs) and meta-analyses, and 10 Level II studies equivalent to RCTs (Appendix 2). In contrast, six non-randomized studies (Level III) and more than 20 observational studies (Level IV) were found, along with several case reports and expert opinions (Levels V-VI). However, many of these studies were small in scale and exhibited wide variability in intervention types, participant age groups, and outcome measures, resulting in a continued scarcity of high-quality evidence. In particular, there is a notable lack of high-quality RCTs that address pediatric populations, comparisons between CMT subtypes, or long-term effects. This highlights the urgent need for multicenter collaborative studies.

Current evidence suggests that exercise therapy (including stretching, aerobic exercise, and resistance training) and orthotic interventions (such as ankle-foot orthoses (AFOs) and orthopedic shoes) may have modest beneficial effects on gait, muscle strength, ADL, balance, and fatigue. However, many reported effects are small and often lack statistical significance. Most studies are small-scale with inconsistent intervention methods and outcome measures, and the evidence levels largely remain within Class II to Class III according to the American Academy of Neurology (AAN) criteria. High-quality studies focusing on pediatric populations and non-ambulatory individuals are particularly lacking, and the development of individually optimized protocols remains a key challenge.

Some aerobic and resistance training programs have shown associations with improvements in VO₂max, sit-to-stand performance, and ADL, whereas nighttime orthotic devices and splints have not demonstrated clear effects on muscle strength or range of motion. Differences in muscle weakness patterns and compensatory movements between CMT subtypes (CMT1/CMT2) have also been reported, underscoring the growing importance of individualized assessments. The adoption of precise, real-world outcome measures, such as 3D gait analysis, plantar pressure evaluation, wearable sensors, and body composition analysis using dual X-ray absorptiometry (DXA), is progressing.

Orthopedic footwear and foot surgery require tailored selection based on the pattern of foot deformity and gait impairment, with timely interventions contributing to functional preservation and improved QOL. These findings strongly support the need for large-scale multicenter RCTs to evaluate the long-term effects of personalized interventions for patients with CMT.

To address this issue, the importance of individualized rehabilitation programs tailored to the progression of symptoms has been increasingly emphasized. The development of technologies capable of objectively and quantitatively evaluating rehabilitation outcomes is essential. Promising directions include gait analysis using artificial intelligence, automated therapeutic interventions, and advanced wearable systems. These innovations are expected to contribute to the establishment of more accurate and optimized management strategies for patients with CMT.

One emerging and increasingly prominent approach is telecoaching (TC), a remote training method that utilizes mobile devices. A recent systematic review has suggested that TC-based training protocols are both effective and safe. However, the number of RCTs remains limited, and the overall methodological quality of existing studies is still insufficient. Therefore, the true efficacy of TC interventions for individuals with CMT has yet to be conclusively determined [36].

Rehabilitation Outcomes in Case Reports

Several case reports suggest the potential benefits of rehabilitation interventions for individuals with CMT:

A 44-year-old male received a 7-week intervention consisting of effleurage, myofascial release, and compression techniques to address low back pain and bilateral foot pain. The treatment resulted in reduced pain and improved range of motion [37].

A 51-year-old male underwent a combined intervention including strength training, orthotic management, and electrical stimulation. He demonstrated improved gait stability and resolution of low back pain. Continued adherence to home-based exercises was also confirmed [31].

A 78-year-old male showed reduced swelling of the left ankle following a comprehensive rehabilitation program [31].

A 16-year-old male with CMT type 1 participated in an 8-month exercise program. Improvements were observed in the 6-minute walk test (6MWT) by 9.3% and in balance testing by up to 15.3% [38].

These cases highlight the potential of tailored rehabilitation approaches to alleviate symptoms and enhance functional outcomes in patients with CMT.

Evidence From Research Studies and Assessment Methods

Research investigating physical activity behaviors and barriers in individuals with CMT has identified fatigue, pain, lack of motivation, and limited time as primary obstacles to sustained physical activity. Continued adherence to strength training appears to be positively influenced by support from healthcare professionals and positive exercise experiences [39].

A double-blind RCT reported that although six months of strength training did not produce significant increases in muscle mass, it contributed to slowing the progression of long-term muscular weakness [40].

For clinical assessment of gait and balance function, the 6MWT, 10-meter walk test (10MWT), and the Short Physical Performance Battery (SPPB) have been shown to be reliable and valid tools. In evaluating foot deformities, the Foot Posture Index (FPI-6) is considered the most suitable measure for individuals with CMT, while other orthopedic scales may not be appropriate for this population [41].

Future research directions

The management of gait impairment in CMT is evolving, yet significant gaps remain in our understanding and assessment of disease progression and therapeutic response. This section outlines key avenues for future research aimed at advancing clinical care and rehabilitation strategies.

Exploration of Novel Biomarkers Related to Gait Impairment

Currently, the evaluation of disease progression in CMT relies predominantly on clinical rating scales and imaging modalities. However, there is a critical lack of validated biological markers (biomarkers) capable of predicting the onset, severity, or progression of gait impairment at an early stage. Future research should focus on the development of objective, predictive models incorporating serum biomarkers, neurophysiological indicators, and gene expression profiles. The integration of such biomarkers could allow for earlier diagnosis, better monitoring of disease progression, and the design of more personalized rehabilitation protocols.

Application of Gait Analysis for Therapeutic Monitoring

Gait dysfunction is one of the most prominent and functionally limiting symptoms in individuals with CMT. As such, gait analysis represents a powerful tool for evaluating the efficacy of therapeutic interventions. Quantitative gait analysis using wearable inertial sensors, pressure-sensitive walkways, or 3D motion capture systems can sensitively detect subtle changes in gait patterns in response to treatment. Future clinical trials and longitudinal studies should prioritize the incorporation of gait metrics as primary outcome measures, thereby improving the objectivity and sensitivity of intervention assessments.

Precision Rehabilitation

The clinical heterogeneity of CMT - driven by variations in genetic subtypes, age of onset, disease severity, and progression rates - presents a major challenge for standardized rehabilitation. To address this, a paradigm shift toward "Precision Rehabilitation" is necessary. This approach involves the integration of multimodal data, including genetic information, neurophysiological findings, and detailed gait analyses, to tailor rehabilitation strategies to the individual. Precision rehabilitation aims to deliver stage-specific and disease-targeted interventions, potentially maximizing functional outcomes and improving patient quality of life.

Standardization of International Evaluation Criteria

Assessment methodologies for gait impairment currently vary widely across clinical and research settings, limiting the ability to compare outcomes and synthesize data across studies. To enhance reproducibility and facilitate multicenter collaborations, the establishment of internationally standardized evaluation protocols is urgently needed. Commonly used measures such as the 6-Minute Walk Test (6MWT), GAITRite system analysis, and consistent gait speed thresholds should be harmonized. Additionally, the incorporation of Patient-Reported Outcome Measures (PROMs) alongside objective performance metrics would provide a more comprehensive evaluation framework that captures both functional status and patient-centered perspectives.

## Conclusions

CMT is a progressive peripheral neuropathy that causes significant gait dysfunction due to muscle weakness, sensory disturbances, and impaired coordination. Gait impairments in individuals with CMT are a serious clinical concern, as they are directly associated with increased risk of falls, reduced ADL, and a substantial decline in QOL.

This review has comprehensively examined the neuropathological basis, clinical features, assessment methods, and therapeutic strategies related to gait impairment in CMT, while outlining key directions for future research. Although limited, the current evidence on rehabilitation was systematically summarized to clarify future perspectives. Exercise therapy and orthotic interventions have shown some potential benefits, but small sample sizes and methodological heterogeneity remain significant limitations. The introduction of gait analysis using artificial intelligence and wearable devices is expected to promote the development of objective evaluation methods. “Precision Rehabilitation,” tailored to genetic subtypes and disease stages, is emerging as a promising approach. There is an urgent need for multicenter RCTs and the international standardization of assessment criteria.

At present, no curative treatment exists for CMT, and care remains largely symptomatic, relying on physical therapy and orthotic interventions. However, standardized rehabilitation protocols have not yet been established. Thus, the development of individualized rehabilitation programs and objective, quantitative gait assessment tools is essential.

Looking ahead, key priorities include the development of novel gait-related biomarkers, clinical application of gait analysis, implementation of precision rehabilitation, and the establishment of internationally standardized evaluation systems. To improve the QOL of individuals with CMT, a multifaceted approach that integrates clinical care, research innovation, and technological advancement is imperative.

## Appendices

Appendix 1

**Table 1:** Detailed search strategy for PubMed (April-May 2025)

Item	Details
Database	PubMed (U.S. National Library of Medicine)
Date of Search	April 15 to May 31, 2025
Search Period	January 1, 1995 to May 31, 2025
Language	English
Species	Humans
Eligible Article Types	Clinical Trial, Randomized Controlled Trial, Controlled Clinical Trial, Observational Study, Systematic Review, Meta-Analysis, Case Series
Exclusion Criteria	Editorials, Letters, Comments, Animal studies, Conference Abstracts
Search Strategy (PubMed)	( "Charcot-Marie-Tooth Disease"[Mesh] OR "Charcot-Marie-Tooth"[tiab] OR "Hereditary Sensory and Motor Neuropathy"[tiab] ) AND ( "Rehabilitation"[Mesh] OR "Exercise Therapy"[Mesh] OR "Physical Therapy Modalities"[Mesh] OR "Orthotic Devices"[Mesh] OR "Ankle Foot Orthosis"[tiab] OR "Gait"[Mesh] OR "Walking Analysis"[tiab] OR "Balance"[Mesh] ) AND ( "1995/01/01"[Date - Publication] : "2025/05/31"[Date - Publication] ) AND (English[lang])
Notes	The search combines MeSH terms and free-text terms to maximize sensitivity. Boolean operators (AND, OR, NOT) are used to combine disease, intervention, and exclusion criteria. Filters were applied to focus on relevant human clinical research.

Appendix 2

**Table 2:** Literature search results

Study design	Year	Authors	Classification
Systematic Reviews/Meta-analyses	2024	Kim et al. [10]	Orthoses and Assistive Devices
	2023	Conde et al. [27]	Exercise and Physical Therapy
	2016	Corrado et al. [9]	Exercise and Physical Therapy
	2015	Sman et al. [8]	Exercise and Physical Therapy
Randomized controlled trials (RCTs)	2024	Dudziec et al. [24]	Exercise and Physical Therapy
	2020	Mori et al. [25]	Exercise and Physical Therapy
	2019	Wallace et al. [26]	Exercise and Physical Therapy
	2014	Ramdharry et al. [28]	Exercise and Physical Therapy
	2006	Refshauge et al. [32]	Orthoses and Assistive Devices
Prospective and retrospective observational studies involving intra-group and inter-group comparisons	2023	Dinesh et al. [35]	Clinical Measures and Biomarkers
	2019	Mori et al. [22]	Clinical Measures and Biomarkers
Case-control studies and cross-sectional studies	2023	Park et al. [21]	Gait, Balance, and Strength Assessment
	2020	Pogemiller et al. [18]	Gait, Balance, and Strength Assessment
Case reports	2024	Philps et al. [30]	Orthoses and Assistive Devices
	2016	Dimitrova et al. [31]	Exercise and Physical Therapy
	2011	Maggi et al. [29]	Exercise and Physical Therapy
	2011	Ferrarin et al. [19]	Clinical Measures and Biomarkers
	2006	Guzian et al. [23]	Orthoses and Assistive Devices
Descriptive studies	2021	Nonnekes et al. [7]	Gait, Balance, and Strength Assessment
	2008	Young et al. [33]	Clinical Measures and Biomarkers
